# FGA-Net: Fourier-guided attention network for feature enhancement in colorectal cancer digital pathology images

**DOI:** 10.3389/fphys.2026.1853691

**Published:** 2026-05-15

**Authors:** Liqun Li, Yiwen Zhang, Kun Wang, Jiandong Tai, Lei Chen

**Affiliations:** 1 The Third Operating Room, The First Hospital of Jilin University, Jilin, Changchun, China; 2 College of Chinese Medicine, Changchun University of Chinese Medicine, Changchun, China; 3 Department of Ophthalmology, The First Hospital of Jilin University, Jilin, Changchun, China; 4 Department of Colorectal and Anal Surgery, General Surgery Center, The First Hospital of Jilin University, Jilin, Changchun, China

**Keywords:** colorectal cancer, digital pathology, feature enhancement, Fourier transform, histopathology image classification

## Abstract

**Introduction:**

Accurate classification of colorectal histopathology images is challenging because diagnostically relevant patterns are distributed across both fine-grained cellular textures and broader tissue architecture. Conventional handcrafted descriptors often lack sufficient discriminative capacity, while purely spatial deep models do not explicitly exploit frequency-domain information.

**Methods:**

We propose FGA-Net, a Fourier-Guided Attention Network for feature enhancement in colorectal cancer digital pathology images. The model combines patch embedding, transformer-style encoding, and a Fourier-guided Attention for feature Refinement (FAR) module integrating FFT/IFFT transformation, squeeze-and-excitation recalibration, and self-attention. Experiments were conducted on the public EBHI dataset under the binary benign-versus-malignant setting at 200× magnification.

**Results:**

FGA-Net consistently outperformed five handcrafted feature families across seven downstream classifiers. The best configuration, FGA-Net with ANN, achieved an accuracy of 87.54%, surpassing the strongest handcrafted baseline by 11.52 percentage points. Ablation studies further showed that both the spectral branch and the channel recalibration branch contributed positively to performance.

**Discussion:**

The results indicate that Fourier-guided attention effectively improves pathology feature representation by jointly modeling spectral cues and long-range spatial dependencies. FGA-Net provides a more discriminative and transferable representation for colorectal histopathology classification and offers a promising direction for future computational pathology research.

## 1 Introduction

Colorectal cancer (CRC) is one of the most common and deadly malignancies worldwide, placing a substantial burden on healthcare systems and cancer screening programs Sung et al. (2021). Histopathological examination remains the gold standard for diagnosis, subtype assessment, and treatment planning, because it provides direct visual evidence of epithelial atypia, glandular disorganization, stromal remodeling, and invasive growth patterns. With the increasing adoption of whole-slide scanners and digital pathology workflows, large collections of pathology images can now be archived, shared, and analyzed computationally, which has accelerated the development of computer-aided diagnosis systems for gastrointestinal pathology Litjens et al. (2017); Campanella et al. (2019). Despite this progress, automatic classification of colorectal pathology images remains challenging. First, colorectal tissue exhibits pronounced intra-class heterogeneity. Even within the same pathological category, the morphology of nuclei, glands, lumen structures, stroma, inflammatory infiltration, and necrotic regions may vary substantially across patients and acquisition conditions. Second, the inter-class boundary can be subtle, especially for samples that differ only in low-grade versus high-grade epithelial atypia or in localized architectural distortion. Third, histopathological images are often affected by stain variation, background regions, tissue folds, blur, and local artifacts, all of which interfere with reliable feature extraction. These challenges are particularly relevant in colorectal digital pathology, where diagnostically meaningful patterns may appear simultaneously at cellular and tissue scales Kather et al. (2019); Hu et al. (2022).

Traditional pathology image classification pipelines largely rely on handcrafted descriptors such as color histograms, local binary patterns, gray-level co-occurrence statistics, and gradient based texture operators. Although these approaches can be computationally lightweight and sometimes effective on relatively simple data, they depend heavily on manually designed priors and typically have limited capacity to encode the complex interplay between local morphology and global tissue organization. In colorectal pathology, where fine nuclear irregularities and larger glandular structures are both diagnostically important, such descriptors often become insufficient. Convolutional neural networks (CNNs) significantly advanced pathology image analysis by learning hierarchical features directly from raw pixels. Architectures such as ResNet and DenseNet have become strong baselines because they model texture and morphology more effectively than handcrafted pipelines He et al. (2016); Huang et al. (2017). However, CNNs remain inherently biased toward local receptive fields. Although deeper layers enlarge the effective context, purely convolutional modeling may still be suboptimal for colorectal pathology, where diagnostically relevant evidence often depends on long-range interactions among tissue regions. Transformer-based vision models address part of this limitation by representing images as token sequences and modeling pairwise relationships through self-attention. Vision Transformer (ViT) and pathology-oriented transformer frameworks have shown strong potential in computational pathology because they naturally capture long-range contextual dependencies Dosovitskiy et al. (2021); Shao et al. (2021); Wang et al. (2022). Nevertheless, most existing transformer-based pathology models operate predominantly in the spatial domain. They rarely exploit frequency-domain priors explicitly, even though histopathological structures such as nuclear boundaries, chromatin texture, lumen contours, and epithelial crowding have characteristic spectral responses.

This observation motivates the present work. In colorectal pathology, high-frequency components often correspond to fine-grained cellular boundaries and detailed textural irregularities, whereas lower-frequency components encode broader tissue arrangement and stain distribution. A feature extractor that explicitly refines token representations in the spectral domain before contextual aggregation may therefore provide a more informative and more transferable representation than purely spatial attention. Inspired by this idea, we propose **FGA-Net**, a **F**ourier-**G**uided Attention Network for feature enhancement in colorectal cancer digital pathology images. The main contributions of this study are summarized as follows. First, we propose a pathology-oriented feature enhancement network that explicitly couples Fourier-domain refinement with transformer-style token interaction. Second, we design a Fourier-guided Attention for feature Refinement (FAR) module that combines FFT/IFFT transformation, squeeze-and-excitation recalibration, and self-attention, enabling the model to enhance diagnostically relevant morphology while suppressing noisy or redundant responses. Third, we provide a comprehensive evaluation of FGA-Net on the public EBHI benchmark and demonstrate that the learned representation consistently outperforms conventional handcrafted descriptors across a diverse set of downstream classifiers.

## 2 Related work

### 2.1 Handcrafted and hybrid feature extraction

Feature extraction has long been the core problem in medical image classification. In early pathology image analysis, descriptors such as color histograms, HOG, LBP, and GLCM were widely used because they provided compact representations and could be combined with simple classifiers. Similar strategies were also adopted in colorectal pathology benchmarks, including the EBHI dataset, where multiple handcrafted descriptors were evaluated under a binary benign-versus-malignant setting Hu et al. (2022). However, handcrafted features are usually sensitive to stain variation, image noise, and morphological diversity, and they often fail to capture the interaction between local cellular texture and larger tissue architecture. To alleviate the limitations of purely manual descriptors, recent work has explored learnable feature refinement mechanisms on top of transformer-based backbones. MIAFEx is a representative example, introducing a token refinement strategy for general medical image classification under limited-data conditions Ramos-Soto et al. (2025). This line of research suggests that lightweight feature refinement can improve transferability and robustness. However, MIAFEx is not specifically designed for colorectal digital pathology and does not explicitly incorporate Fourier-domain priors, which are highly relevant for tissue texture and glandular boundary modeling. Our method extends this direction by performing explicit spectral refinement before contextual attention.

### 2.2 CNN-based digital pathology classification

CNNs have become the dominant paradigm in computational pathology because of their ability to learn hierarchical visual features directly from data. Residual and densely connected architectures, represented by ResNet and DenseNet, have been widely adopted as strong medical image backbones He et al. (2016); Huang et al. (2017). In colorectal pathology, deep learning has enabled improved recognition of morphological patterns that are difficult to encode manually. However, CNNs mainly rely on repeated local convolutions, which makes them highly effective for local texture modeling but less direct for long-range relational reasoning across distant tissue regions.

### 2.3 Transformer-based pathology modeling

Transformers complement CNNs by modeling image patches as token sequences and learning pairwise token interaction via self-attention. ViT established that pure transformer architectures can achieve strong performance in visual recognition tasks Dosovitskiy et al. (2021). In pathology, transformer-based models such as TransMIL and CTransPath have further demonstrated the value of global context aggregation and transferable pretraining for histopathological representation learning Shao et al. (2021); Wang et al. (2022). Even so, most of these models remain fundamentally spatial in formulation, and their ability to emphasize frequency-sensitive pathology cues is implicit rather than explicit.

### 2.4 Frequency-aware representation learning

Frequency-domain modeling has recently attracted increasing attention as a complementary strategy for visual representation learning. Fast Fourier Convolution showed that spectral operations can provide efficient global information mixing and non-local receptive fields Chi et al. (2020). In parallel, squeeze-and-excitation networks demonstrated that adaptive channel recalibration can selectively emphasize informative channels while suppressing irrelevant ones Hu et al. (2018). These ideas are particularly appealing in pathology, where subtle textural and structural changes often correspond to meaningful spectral signatures. FGA-Net builds on this intuition by integrating frequency-domain enhancement and channel-wise recalibration inside a transformer-style encoder designed for colorectal pathology.

## 3 Methods

### 3.1 Problem formulation

Let $D={\left\{\left(x_{i},y_{i}\right)\right\}}_{i=1}^{M}$ denote a colorectal pathology dataset, where $x_{i}\in {\mathbb{R}}^{3\times H\times W}$ is an RGB image tile and $y_{i}\in \left\{1,\ldots ,C\right\}$ is its class label. The goal is to learn a mapping expressed as Equation 1:

(1) $$f_{\theta }:{\mathbb{R}}^{3\times H\times W}\to {\left[0,1\right]}^{C}$$

that predicts the posterior probability distribution over pathological classes. In this work, each input image is resized to 224 × 224 before being processed by the network.

### 3.2 Overall architecture

**Figure 1:**
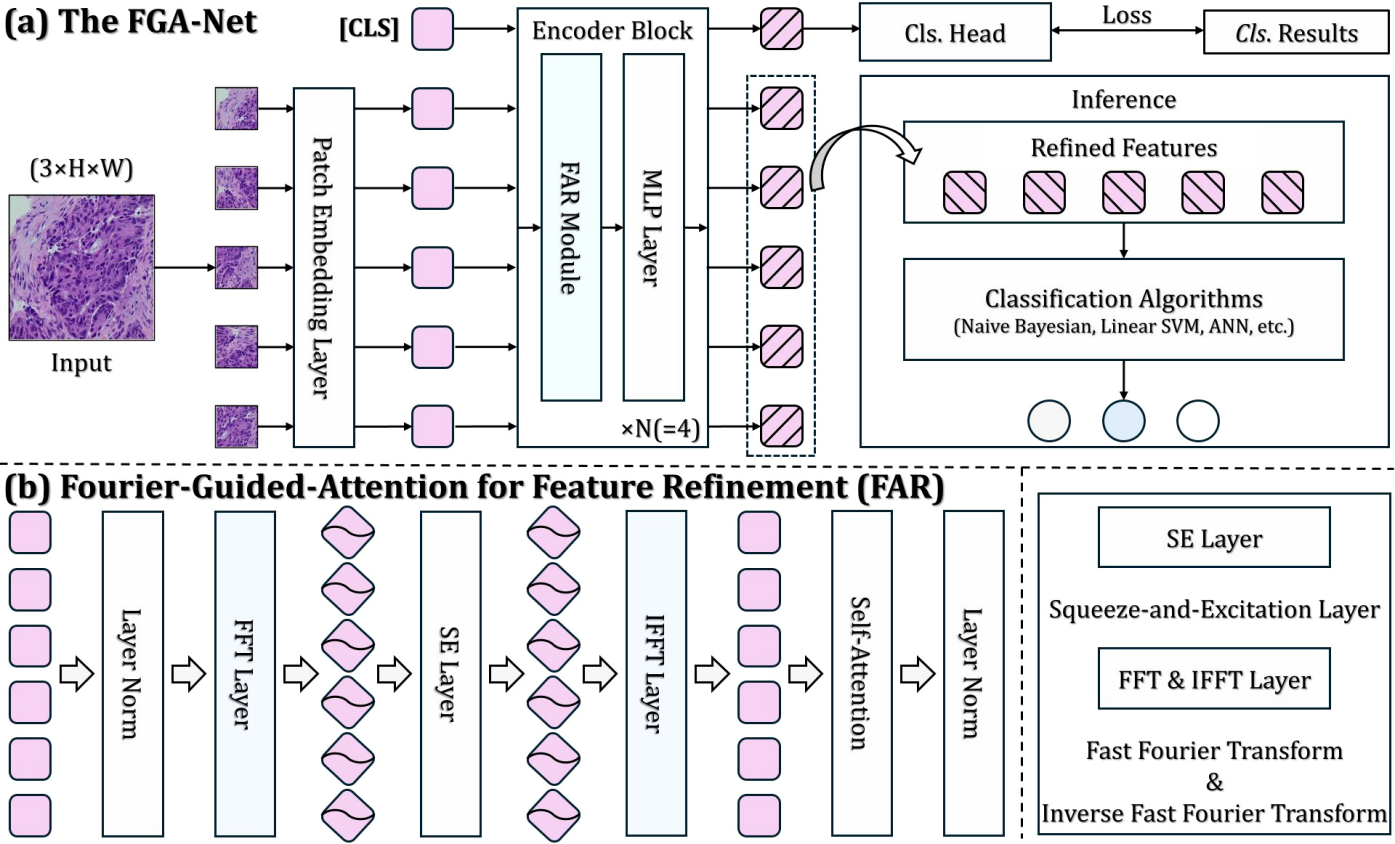
**(a)** Overall architecture of the proposed FGA-Net. The network first tokenizes the colorectal pathology image through patch embedding and then refines the token sequence using stacked encoder blocks. **(b)** Each encoder block consists of a FAR, Fourier-guided Attention for feature Refinement module and an MLP layer. During training, the final class token is fed into a classification head, while during inference the refined representation can also be exported and evaluated by shallow classifiers.

**Figure 2:**
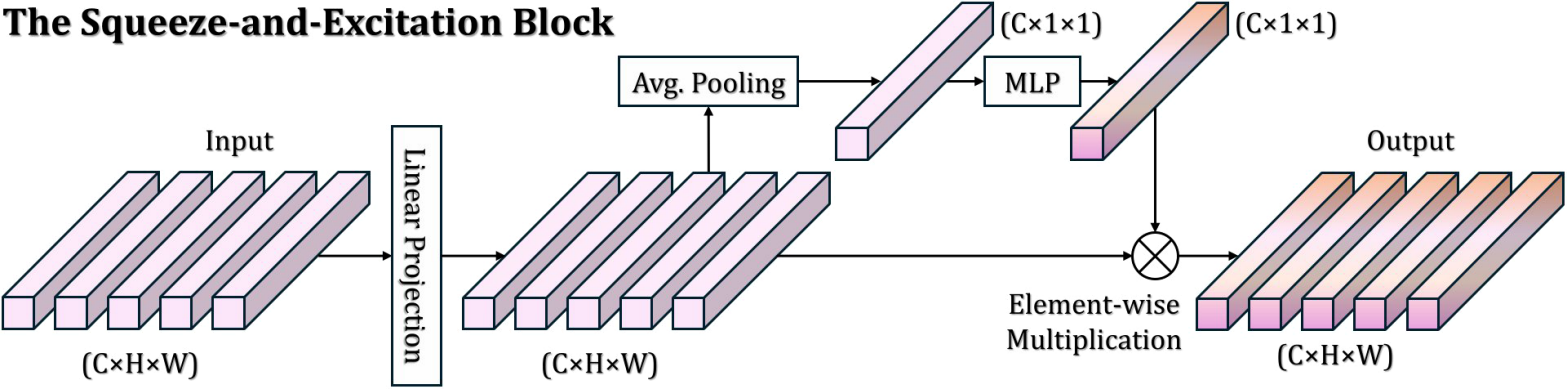
Structure of the SE, squeeze-and-excitation block. The input feature is first compressed through global information aggregation, then passed through a lightweight gating branch to learn channel-wise importance weights, and finally rescaled to emphasize informative channels while suppressing less relevant responses.

The architecture of the proposed FGA-Net is shown in **Figure 1**. Given an input pathology image, the network first divides it into non-overlapping patches and maps them into token embeddings. A learnable classification token is prepended to the patch sequence, and the resulting token set is processed by a stack of encoder blocks. Each encoder block contains a Fourier-guided Attention for feature Refinement (FAR) module followed by an MLP layer. The FAR module is the core component of FGA-Net. It first projects patch tokens into the frequency domain using FFT, then recalibrates spectral channels through a squeeze-and-excitation mechanism (**Figure 2**), reconstructs the refined token representation through IFFT, and finally applies self-attention to model long-range contextual dependencies. In this way, the model jointly exploits spectral priors and token interaction. During training, the final class token is fed to an internal classification head and optimized using cross-entropy loss. During inference, FGA-Net can be used in two ways. It can either act as an end-to-end classifier through the internal head or operate as a feature extractor, where the refined representation is exported and evaluated by external shallow classifiers such as LR, *k*NN, SVM, RF, and ANN. The latter protocol is used in the present study because our primary focus is the quality and transferability of the extracted feature representation.

### 3.3 Patch embedding and token initialization

Given an input image $x\in {\mathbb{R}}^{3\times H\times W}$, we divide it into non-overlapping patches of size *P* × *P*, where *P* = 16. The total number of patches is expressed as Equation 2:

(2) $$N=\frac{H}{P}\times \frac{W}{P}.$$

For H=W=224, this yields N=196 patch tokens. Each patch is flattened and linearly projected into a *D*-dimensional embedding space, with D=768 in our implementation. Let $X_{p}\in {\mathbb{R}}^{N\times D}$ denote the patch embedding matrix. A learnable class token $z_{\text{cls}}\in {\mathbb{R}}^{1\times D}$ is prepended to the token sequence, and learnable positional embeddings $E_{\text{pos}}$ are added (Equation 3):

(3) $$Z^{0}=\left[z_{\text{cls}};X_{p}\right]+E_{\text{pos}},\;\;Z^{0}\in {\mathbb{R}}^{\left(N+1\right)\times D}.$$

In this way, the input pathology image is converted into an ordered token representation that jointly preserves local patch content, global image-level summarization through the class token, and spatial arrangement through positional encoding. This initialization provides the foundation for subsequent Fourier-guided refinement and long-range contextual modeling in the encoder.

### 3.4 Fourier-guided attention for feature refinement

The core motivation of the proposed FAR module is that colorectal histopathology images contain diagnostically relevant information in both the spatial and frequency domains. In the spatial domain, the network should preserve tissue layout, gland arrangement, and contextual relationships among different image regions. In the frequency domain, however, many subtle pathological cues are expressed more clearly through spectral responses, such as nuclear boundary sharpness, chromatin irregularity, lumen contour variation, epithelial crowding, and textural disorder caused by dysplasia or invasive growth. A purely spatial attention mechanism can capture long-range interactions among image patches, but it does not explicitly emphasize those spectral patterns. For this reason, we introduce a Fourier-guided refinement process before self-attention, so that the token representation is first enhanced from a spectral perspective and then globally aggregated in the token space.

Specifically, given the input token sequence of the (*l* − 1)-th encoder block, we separate the class token from the patch tokens and reshape the patch-token matrix back to its two-dimensional patch layout. This step is important because the Fourier transform should operate on a spatially structured representation rather than on an unordered token list. After layer normalization, the reshaped feature map is transformed into the frequency domain by a two-dimensional fast Fourier transform. Instead of treating all spectral channels equally, FAR further introduces a channel-adaptive recalibration mechanism. The transformed response is summarized channel-wise and fed into a squeeze-and-excitation branch, which learns to highlight informative spectral channels and suppress irrelevant ones. In colorectal pathology, this operation is particularly useful because not all spectral responses are equally meaningful: some channels may capture discriminative micro-textures and glandular boundaries, while others may be dominated by stain fluctuation, background variation, or acquisition noise. Once the channel-wise importance has been estimated, the recalibrated spectral representation is projected back to the spatial domain through the inverse Fourier transform. The resulting feature map is then added to the original patch tokens through a residual connection. In this way, FAR does not discard the original spatial representation; instead, it enriches the original tokens with frequency-aware enhancement. This residual design also stabilizes optimization and avoids excessive distortion of the learned pathology structure. After the spectral refinement stage, the enhanced patch tokens are concatenated again with the class token and passed to the multi-head self-attention layer. The role of self-attention here is to propagate the refined morphological cues across the entire image, allowing distant but related tissue regions to interact directly. This is desirable for colorectal pathology, where diagnostically meaningful evidence often depends not only on local nuclear atypia but also on how multiple epithelial and stromal regions are organized within the image. The overall FAR operation can therefore be summarized as Equation 4:

(4) $$U^{l-1}=T^{l-1}+\text{IFFT}\left(G(\text{FFT }(\text{LN(}T^{l-1}\text{))})\right),$$

where **T***^l^*^−1^ denotes the patch-token representation, FFT(·) and IFFT(·) are the forward and inverse Fourier transforms, and G(·) represents the SE-based channel recalibration in the spectral domain. The refined tokens are then integrated with the class token and processed by self-attention (Equation 5):

(5) $${\bar{Z}}^{l}=Z_{f}^{l-1}+\text{MSA }\left(\text{LN}\left(Z_{f}^{l-1}\right)\right),\;Z_{f}^{l-1}=\left[z_{\text{cls}}^{\;l-1};U^{l-1}\right].$$

Compared with a standard transformer block, FAR introduces an explicit spectral enhancement stage before contextual token interaction. This design gives the encoder two complementary capabilities. First, the Fourier branch increases sensitivity to frequency-sensitive pathology morphology, especially subtle textural and boundary-related abnormalities. Second, the self-attention branch preserves the transformer’s ability to model long-range spatial dependency across tissue regions. By combining these two mechanisms within one refinement block, FGA-Net can better capture the multi-scale and heterogeneous nature of colorectal pathology images.

### 3.5 Encoder block and classification objective

After the FAR module completes frequency-guided enhancement, the refined token sequence is further processed by a feed-forward transformation to improve nonlinear representation capacity. Concretely, each encoder block in FGA-Net follows a transformer-style residual structure in which the output of the self-attention branch is passed through a multi-layer perceptron (MLP) with layer normalization and skip connection. This stage allows the network to further reorganize and project the refined pathology representation into a more discriminative latent space. While the FAR module mainly emphasizes informative spectral cues and long-range token interaction, the MLP layer serves as an additional feature transformation unit that strengthens channel mixing and improves the expressiveness of the encoder block. The output of the *l*-th encoder block is formulated as Equation 6:

(6) $$Z^{l}={\bar{Z}}^{l}+\text{MLP }\left(\text{LN}\left({\bar{Z}}^{l}\right)\right),$$

where ${\bar{Z}}^{l}$ is the output of the FAR-guided self-attention branch. In our implementation, the MLP consists of two fully connected layers with GELU activation, and its hidden dimension is expanded to four times the embedding dimension. By stacking four such encoder blocks, FGA-Net progressively refines the pathology representation from shallow visual patterns to more abstract and task-relevant morphology. This progressive refinement is particularly important for colorectal histopathology, where diagnostically relevant evidence may range from subtle local nuclear atypia to larger glandular disorganization and tissue-level structural distortion.

After the final encoder block, the class token is extracted as the global image representation. This token aggregates information from all pathology patches through repeated spectral refinement, self-attention interaction, and nonlinear transformation, and therefore serves as a compact summary of the entire image. For end-to-end training, the final class token is passed through a linear classification head followed by a softmax function to produce the predicted probability of each class (Equation 7):

(7) $$\hat{y}=\text{Softmax }\left(W_{c}z_{\text{cls}}^{L}+b_{c}\right),$$

where $z_{\text{cls}}^{L}$ denotes the class token from the last encoder block, and $W_{c}$ and $b_{c}$ are the parameters of the classifier. The training objective is the standard cross-entropy loss, which is formulated as Equation 8:

(8) $${ℒ}_{\text{CE}}=-\overset{C}{\underset{c=1}{\sum }}y_{c}\text{log }{\hat{y}}_{c}$$

where *y_c_* and ${\hat{y}}_{c}$ denote the ground-truth label and predicted probability for class *c*, respectively. This objective encourages the network to assign high confidence to the correct pathological category and low confidence to the others. Since the class token is optimized jointly with the spectral refinement and attention modules, the learned representation gradually becomes more aligned with the decision boundary required for colorectal pathology classification.

It should be noted that FGA-Net is designed not only as an end-to-end classifier but also as a transferable feature extractor. In addition to using the internal classification head, the final class token can be directly exported as a refined pathology descriptor for downstream shallow classifiers. This design is consistent with the evaluation protocol adopted in this study, where the quality of the learned representation is examined under multiple classifiers such as LR, *k*NN, RF, SVM, and ANN. Therefore, the classification objective plays a dual role: it optimizes the end-to-end discrimination ability of the network while simultaneously shaping a more robust and transferable feature space for external classification.

## 4 Experiments

### 4.1 Datasets

**Figure 3:**
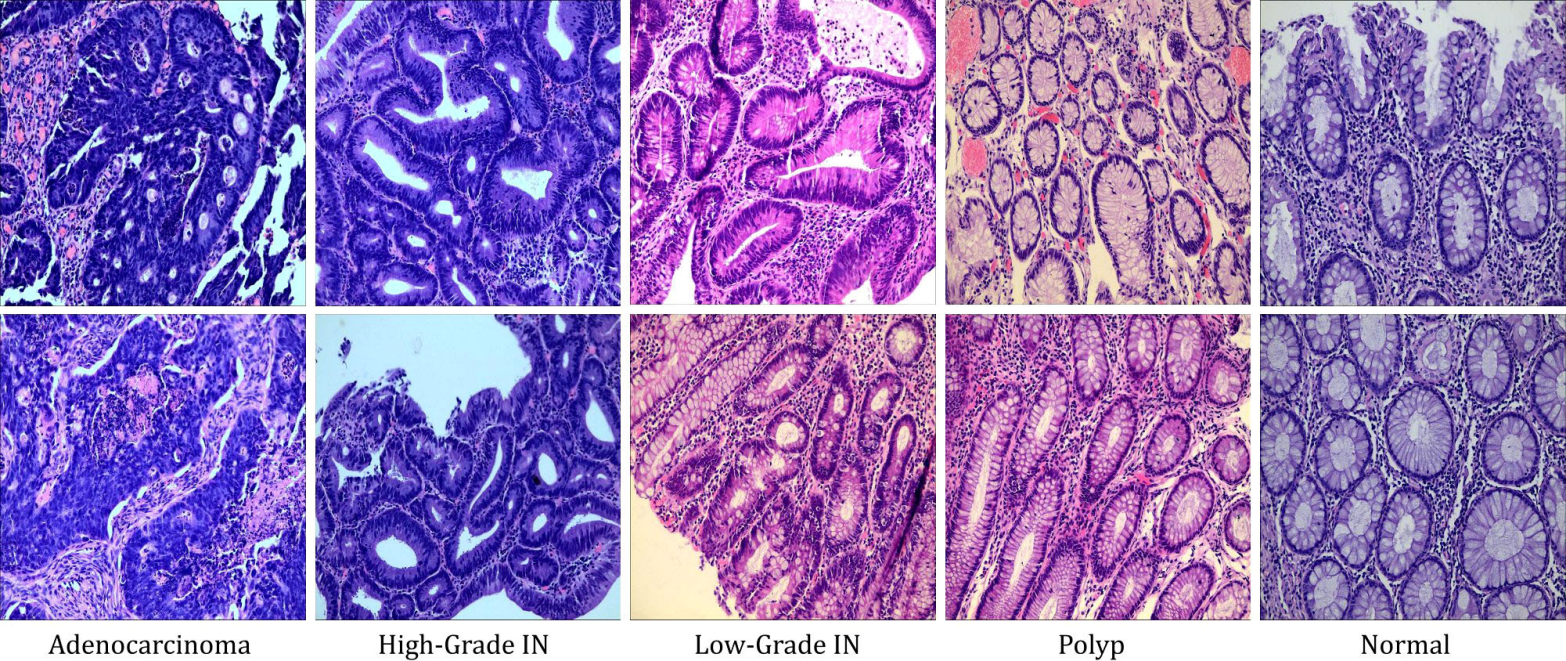
Several samples of the analyzed EBHI dataset. From left to right, the tissue samples correspond to adenocarcinoma, high-grade IN, intraepithelial neoplasia, low-grade IN, intraepithelial neoplasia, hyperplastic polyp, and normal mucosa.

All experiments were conducted on the public Enteroscope Biopsy Histopathological H&E Image (EBHI) dataset Hu et al. (2022). EBHI contains 5,532 H&E-stained enteroscope biopsy images collected at four magnifications (40×, 100×, 200×, and 400×) and five pathological categories: Normal, Polyp, Low-grade Intraepithelial Neoplasia (Low-grade IN), High-grade Intraepithelial Neoplasia (High-grade IN), and Adenocarcinoma. **Figure 3** shows some examples of the five categories. All images were acquired using an Olympus microscope, stored in PNG format, and released at a native resolution of 2048 × 1536. Following the benchmark setting used in the EBHI paper and consistent with the provided experimental results, we adopted the 200× subset for all experiments. As shown in **Table 1**, this subset contains 1,838 images in total and provides an appropriate balance between cellular detail and tissue architecture. To align the task with a clinically meaningful binary classification setting, the original five categories were grouped into two super-classes: Benign (Normal, Polyp, and Low-grade IN) and Malignant (High-grade IN and Adenocarcinoma). This yields 918 benign images and 920 malignant images, resulting in an almost perfectly balanced binary benchmark.

**Table 1:** Summary of the EBHI 200× subset and the binary classification protocol used in this study.

Category	Images at 200 ×	Pathological meaning	Binary label
Normal	61	Histologically normal colorectal mucosa	Benign
Polyp	254	Non-invasive polypoid lesion	Benign
Low-grade IN	603	Mild-to-moderate epithelial dysplasia	Benign
High-grade IN	130	Severe epithelial dysplasia	Malignant
Adenocarcinoma	790	Invasive colorectal carcinoma	Malignant
Total	**1,838**	H&E enteroscope biopsy images	**918/920**

The bold values indicate the total number of images and labels.

### 4.2 Implementation details

All images were resized to 224 × 224 before feature extraction or network training. For FGANet, we applied pathology-preserving online augmentation during training, including random horizontal and vertical flips, 90°C rotations, mild color jitter, and random resized cropping. These operations improve robustness to orientation changes and stain fluctuation while preserving diagnostic semantics. To ensure a fair comparison, the handcrafted baselines followed the feature families reported in the EBHI benchmark, namely color histogram, luminance histogram, HOG, LBP, and GLCM Hu et al. (2022). Each feature family was evaluated using seven shallow classifiers: logistic regression (LR), *k*-nearest neighbors (*k*NN), Naive Bayes, random forest (RF), linear SVM, non-linear SVM, and artificial neural network (ANN). Following the EBHI setting, *k*NN used *k* = 9, RF used 10 trees, the non-linear SVM employed an RBF kernel, and the ANN used a two-hidden-layer architecture. For FGA-Net, the input image was partitioned into 16 × 16 patches, producing 196 patch tokens. The embedding dimension was set to 768, the number of encoder blocks was set to 4, and each FAR module combined FFT/IFFT transformation, SE recalibration, and self-attention. The network was trained for 100 epochs using AdamW with an initial learning rate of 1 × 10^−4^, a weight decay of 5 × 10^−2^, and a batch size of 32. After training, the refined class token was exported and evaluated using the same seven shallow classifiers as the handcrafted baselines. This experimental design, conceptually similar to the feature-oriented evaluation philosophy in MIAFEx Ramos-Soto et al. (2025), allows us to assess the discriminative quality of the representation itself rather than conflating representation learning with a specific end-to-end classifier head. We report Accuracy, class-wise Precision, Recall, Specificity, and F1-score, all in percentage (%). Since the benign and malignant categories are nearly balanced, accuracy remains informative, while class-wise metrics further reveal whether a model is biased toward one class.

### 4.3 Comparison with conventional descriptors and FGA-Net features

**Table 2** presents the complete quantitative comparison between five handcrafted feature families and the proposed FGA-Net representation under the same seven downstream classifiers. Several clear patterns emerge. The proposed FGA-Net features outperform all handcrafted descriptors for every downstream classifier. The best overall configuration is FGA-Net + ANN, which reaches 87.54% accuracy and exceeds the strongest handcrafted baseline (HOG + ANN, 76.02%) by 11.52 percentage points. Even when coupled with simpler classifiers, FGA-Net remains highly competitive. For instance, the LR accuracy improves from the best handcrafted 67.57% to 76.41%, the *k*NN accuracy improves from 72.21% to 79.18%, and the linear SVM accuracy improves from 55.86% to 62.16%. These results indicate that the representation learned by FGA-Net is not only more discriminative, but also easier to separate using both linear and non-linear decision boundaries.

**Table 2:** Complete comparison of handcrafted feature families reported in Hu et al. (2022) and the proposed FGA-Net representation.

Feature family	Classifier	Acc.	Malignant				Benign			
			Prec.	Recall	Spec.	F1	Prec.	Recall	Spec.	F1
CH	LR	51.23	51.41	49.46	53.01	50.42	51.05	53.01	49.46	52.01
	*k*NN	68.94	70.83	64.67	73.22	67.61	67.34	73.22	64.67	70.16
	Naive Bayes	59.67	69.57	34.78	84.70	46.38	56.36	84.70	34.78	67.69
	RF	**73.57**	69.68	83.70	63.39	76.05	79.45	63.39	83.70	70.52
	Linear SVM	55.04	59.22	33.15	77.05	42.51	53.41	77.05	33.15	63.09
	Non-linear SVM	50.14	50.14	100.00	0.00	66.79	50.14	100.00	0.00	66.79
	ANN	70.03	70.11	70.11	69.95	70.11	69.95	69.95	70.11	69.95
LH	LR	57.22	57.54	55.98	58.47	56.75	56.91	58.47	55.98	57.68
	*k*NN	72.21	74.70	67.39	77.05	70.86	70.15	77.05	67.39	73.44
	Naive Bayes	57.49	62.07	39.13	75.96	48.00	55.38	75.96	39.13	64.06
	RF	**72.75**	68.26	85.33	60.11	75.85	80.29	60.11	85.33	68.75
	Linear SVM	53.41	52.79	66.85	39.89	58.99	54.48	39.89	66.85	46.06
	Non-linear SVM	50.14	50.14	100.00	0.00	66.79	-	0.00	100.00	0.00
	ANN	68.94	68.23	71.20	66.67	69.68	69.71	66.67	71.20	68.16
HOG	LR	67.57	67.57	67.93	67.21	67.75	67.58	67.21	67.93	67.40
	*k*NN	66.21	66.30	66.30	66.12	66.30	66.12	66.12	66.30	66.12
	Naive Bayes	60.76	68.87	39.67	81.97	50.34	57.47	81.97	39.67	67.57
	RF	64.31	62.33	72.83	55.74	67.17	67.11	55.74	72.83	60.90
	Linear SVM	55.86	54.82	67.93	43.72	60.68	57.55	43.72	67.93	49.69
	Non-linear SVM	50.14	50.14	100.00	0.00	66.79	-	0.00	100.00	0.00
	ANN	**76.02**	73.76	80.98	71.04	77.20	78.79	71.04	80.98	74.71
LBP	LR	**65.67**	68.35	58.70	72.68	63.16	63.64	72.68	58.70	67.86
	*k*NN	55.86	55.85	57.07	54.64	56.45	55.87	54.64	57.07	55.25
	Naive Bayes	53.95	54.39	50.54	57.38	52.39	53.57	57.38	50.54	55.41
	RF	56.95	56.25	63.59	50.27	59.69	57.86	50.27	63.59	53.80
	Linear SVM	48.50	48.54	45.11	51.91	46.76	48.47	51.91	45.11	50.13
	Non-linear SVM	50.14	50.14	100.00	0.00	66.79	-	0.00	100.00	0.00
	ANN	61.04	61.33	60.33	61.75	60.82	60.75	61.75	60.33	61.25
GLCM	LR	62.67	67.41	49.46	75.96	57.05	59.91	75.96	49.46	66.99
	*k*NN	64.03	63.68	65.76	62.30	64.71	64.41	62.30	65.76	63.33
	Naive Bayes	57.22	61.74	38.59	75.96	47.49	55.16	75.96	38.59	63.91
	RF	60.76	59.71	66.85	54.64	63.08	62.11	54.64	66.85	58.14
	Linear SVM	55.86	71.15	20.11	91.80	31.36	53.33	91.80	20.11	67.47
	Non-linear SVM	56.95	66.25	28.80	85.25	40.15	54.36	85.25	28.80	66.38
	ANN	**65.67**	67.68	60.33	71.04	63.79	64.04	71.04	60.33	67.36
**FGA-Net**	LR	76.41	77.15	75.32	78.64	76.12	78.29	78.87	75.45	78.53
	*k*NN	79.18	80.24	78.56	81.33	79.37	81.14	81.65	78.71	81.22
	Naive Bayes	77.82	83.19	72.48	88.51	77.63	76.94	88.75	72.36	82.38
	RF	83.47	84.52	86.11	81.93	85.26	82.68	81.79	86.44	84.17
	Linear SVM	62.16	61.07	63.58	72.43	62.25	64.77	72.61	63.84	68.42
	Non-linear SVM	65.31	66.05	65.73	64.92	65.89	65.46	64.88	65.67	65.19
	ANN	**87.54**	88.35	89.12	86.76	88.68	87.23	86.59	89.41	87.95

Best accuracy within each feature family is shown in bold. All values are reported in %. CH, Color histogram, LH, Luminance histogram.

The bold values indicate the best result within each feature family.

**Figure 4:**
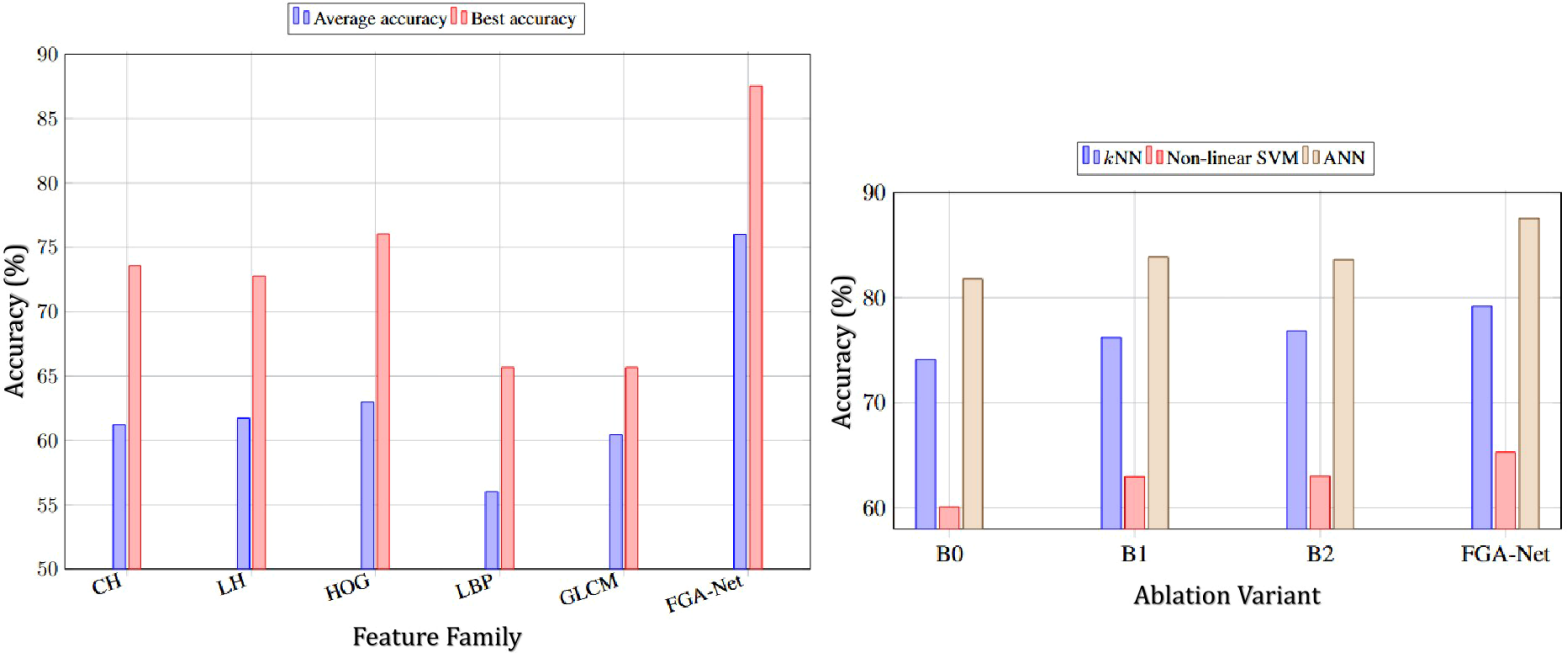
Performance evaluation of the proposed framework. (Left) Comparison of the FGA method against traditional feature families (Color, Lum, HOG, LBP, GLCM), where FGA achieves superior average and best accuracy. (Right) Ablation study of FGA-Net variants (B0, B1, B2), demonstrating consistent performance gains with the full model reaching peak accuracy near 88%.

Additionally, the gain is robust across classifiers rather than concentrated in a single setting. As shown in the left side of **Figure 4**, FGA-Net consistently dominates the handcrafted feature families in both average accuracy and best-case accuracy. This suggests that the proposed representation substantially reshapes the feature space and makes it more compatible with margin-based classification.

### 4.4 Ablation study

To quantify the contribution of the core components in the FAR module, we designed an ablation study with four variants: B0, a baseline encoder without FFT/IFFT and without SE recalibration; B1, a model with FFT/IFFT enabled but without SE; B2, a model with SE enabled but without FFT/IFFT; and the full FGA-Net, which combines both components. **Table 3** and the right side of **Figure 4** show that both components contribute positively. Compared to the baseline B0, introducing the frequency branch alone (B1) improves the accuracy from 74.09% to 76.22% for *k*NN, from 60.11% to 62.98% for non-linear SVM, and from 81.79% to 83.86% for ANN. Using the SE recalibration branch alone (B2) yields similarly consistent gains. However, the best result is always achieved when both modules are activated simultaneously. The full FGA-Net reaches 79.18%, 65.31%, and 87.54% under *k*NN, non-linear SVM, and ANN, respectively. These results indicate that the spectral branch and the channel recalibration branch are complementary rather than redundant.

**Table 3:** Ablation study of the proposed FAR module.

Model	FFT&IFFT	SE	Accuracy (%)		
			*k*NN	Non-linear SVM	ANN
B0	×	×	74.09	60.11	81.79
B1	✓	×	76.22	62.98	83.86
B2	×	✓	76.83	63.02	83.61
FGA-Net	✓	✓	**79.18**	**65.31**	**87.54**

The full FGA-Net combines both components. B0 denotes the baseline model without the FFT/IFFT branch and without the SE recalibration mechanism, B1 includes the FFT/IFFT branch only, and B2 includes the SE mechanism only.

The bold values indicate the best result among the models.

## 5 Discussion

The experimental results consistently show that FGA-Net provides a substantially more informative representation for colorectal pathology classification than conventional handcrafted descriptors. While handcrafted features can still perform acceptably under carefully matched classifiers, their performance is unstable and highly feature-dependent. By contrast, FGA-Net improves performance for every tested classifier, which suggests that the feature space learned by the proposed model is intrinsically more discriminative and more transferable. This advantage can be explained by the design of the FAR module. Handcrafted features mainly encode local gradients, intensity statistics, or predefined textural regularities. Such cues are often insufficient for colorectal pathology images characterized by heterogeneous glands, irregular nuclei, stromal variability, and stain-induced appearance shifts. FGA-Net addresses these limitations by explicitly modeling spectral responses through FFT/IFFT transformation, adaptively reweighting discriminative channels through SE recalibration, and propagating the enhanced information globally through self-attention. The ablation results confirm that both the frequency-domain branch and the channel recalibration branch are individually useful and jointly more effective. At the same time, the present work should be interpreted in the context of its experimental design. The original EBHI benchmark reported a best end-to-end classification accuracy of 95.37% using VGG16 Hu et al. (2022), which is higher than the 87.54% achieved here by *FGA-Net + ANN*. However, the two settings serve different purposes. The EBHI benchmark primarily evaluates complete end-to-end classifiers, whereas the present study focuses on *feature extractor quality and transferability* under a unified shallow-classifier protocol. From this perspective, the more relevant comparison is against alternative feature families under the same downstream classifiers, and under this comparison FGA-Net consistently achieves the best performance. This distinction is important, because transferable feature extraction is valuable in practical scenarios where lightweight classifiers, hybrid pipelines, or downstream analytics are preferred. Several limitations should also be acknowledged. First, the present study uses only the 200× binary subset of EBHI and does not yet explore the full multi-class or multi-magnification setting. Second, our experiments are image-level rather than whole-slide-level, and therefore do not address weakly supervised slide aggregation or lesion localization. Third, although the results on EBHI are strong, external validation on multi-center colorectal pathology datasets remains necessary to assess cross-domain robustness. Future work can extend FGA-Net toward multi-scale pathology modeling, external-domain adaptation, and end-to-end slide-level colorectal diagnosis.

## 6 Conclusion

This study presented FGA-Net, a Fourier-guided attention network for feature enhancement in colorectal cancer digital pathology images. By combining spectral refinement, channel-wise recalibration, and self-attention-based context modeling, FGA-Net learns a transferable pathology representation that is substantially stronger than conventional handcrafted descriptors on the EBHI binary classification benchmark. Under a unified downstream-classifier protocol, the proposed representation achieved consistent gains across all seven classifiers, with the best configuration reaching 87.54% accuracy and balanced malignant and benign F1-scores. The ablation study further demonstrated that the FFT/IFFT pathway and the SE branch are both beneficial and jointly complementary. Overall, the results validate Fourier-guided attention as a promising design principle for colorectal pathology representation learning and provide a strong basis for future work on multi-scale and whole-slide computational pathology.

## Data Availability

The dataset analyzed for this study can be found in https://doi.org/10.6084/m9.figshare.16999363.v1.
